# Aldh1l1‐Cre/ERT2 Drives Flox‐Mediated Recombination in Peripheral and CNS Infiltrating Immune Cells in Addition to Astrocytes During CNS Autoimmune Disease

**DOI:** 10.1002/brb3.70239

**Published:** 2025-02-05

**Authors:** Mario Amatruda, Juan Turati, Josh Weiss, Jorge Villavicencio, Zhihong Chen, Graham Britton, Sam Horng

**Affiliations:** ^1^ Department of Neurology Icahn School of Medicine at Mount Sinai New York New York USA; ^2^ Department of Genetics and Genomics Sciences Icahn School of Medicine at Mount Sinai New York New York USA; ^3^ Department of Immunology and Immunotherapy Icahn School of Medicine at Mount Sinai New York New York USA

**Keywords:** Aldh1l1‐Cre, astrocyte‐specific promoter, central nervous system (CNS) autoimmune disease, CNS infiltrating immune cell, experimental autoimmune encephalomyelitis (EAE), flow cytometry, Gfap‐Cre

## Abstract

**Introduction:**

The transgenic murine *Cre/loxP* system is deployed to investigate the role of central nervous system (CNS) cell‐specific gene alterations in both healthy conditions and models of neurologic disease. The *Aldh1l1‐Cre/ERT2* line is widely used to target astrocytes with high coverage and specificity within the CNS. Specificity outside the CNS, however, has not been well‐characterized, and Aldh1l1‐Cre/ERT2‐mediated recombination within the spleen has been reported. In many CNS diseases, infiltrating immune cells from the periphery drive or regulate pathogenesis. We tested whether flox‐mediated recombination from Aldh1l1‐Cre/ERT2 occurs in immune cells in addition to astrocytes and whether these cells traffic from the spleen into the spinal cord during experimental autoimmune encephalomyelitis (EAE), a model of CNS autoimmune disease.

**Methods:**

Two astrocyte‐targeted mouse lines were generated with the red fluorescent reporter, tdTomato, by crossing the Cre‐recombinase lines, *Tg(Aldh1l1‐Cre/ERT2)1Khakh* and *Tg(Gfap‐Cre)73.12Mvs*, with the reporter line, *Gt(ROSA)26Sor*. Aldh1l1‐Cre/ERT2 was activated with 5 days of intraperitoneal tamoxifen, whereas Gfap‐Cre was constitutively active. EAE was induced 2 weeks after tamoxifen, and then spleens and spinal cords were harvested and processed for flow cytometry at various time points after disease onset in EAE versus healthy controls.

**Results:**

In EAE, Aldh1l1‐Cre/ERT2, but not Gfap‐Cre, induced multiple tdTomato^+^ immune cell subpopulations in the spleen and spinal cord, including macrophages, monocytes, neutrophils, eosinophils, B cells, CD4^+^, and CD8^+^ T cells.

**Conclusion:**

Use of Aldh1l1‐Cre/ERT2 should therefore account for recombination in both astrocytes and immune cells in disease models involving peripheral immune cell infiltration into the CNS.

## Graphical abstract

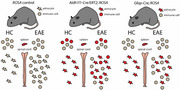

## 1 Introduction

Astrocytes are among the most numerous resident cells of the central nervous system (CNS) (von Bartheld, Bahney, and Herculano‐Houzel 2016) and regulate a diverse array of critical homeostatic and disease‐related processes (Verkhratsky et al. 2023), including processes of injury and repair during neuroinflammation (Han et al. 2021; Liddelow et al. 2017; Linnerbauer, Wheeler, and Quintana 2020; Sanmarco, Polonio et al. 2021; Sanmarco, Wheeler et al. 2021; Wheeler et al. 2020), ischemia (Hernandez et al. 2023; Ito et al. 2019; Prescott et al. 2023), neurodegeneration (Guttenplan et al. 2021), and trauma (Herrmann et al. 2008; Sofroniew 2021). Crosstalk between astrocytes and other cell types within the CNS involves both resident cells (microglia, neurons, among others) and peripheral infiltrating immune cells, tuning CNS functions in a dynamic, regional, and context‐specific manner (Burda et al. 2022; Hasel et al. 2021; Sofroniew 2020; Williams et al. 2020). Decoding these molecular crosstalk pathways is likely to identify novel translational targets against neurologic disease (Han et al. 2021; Kiss et al. 2023).

To study these processes, astrocyte‐specific promoter‐driven Cre‐recombinases are used in combination with loxP‐modified genes to downregulate or upregulate functional proteins and regulatory RNAs in developmental, healthy, or disease‐related contexts of interest. Functional interpretation of these studies relies upon the specificity and coverage of the promoter within the experimental system. Multiple constitutive and inducible promoter‐driven Cre‐recombinase lines targeting astrocytes have been engineered, including several mGfap‐Cre, hGFAP‐Cre, GLAST‐Cre, S100b‐Cre, Cx30‐Cre, and Aldh1l1‐Cre lines, which have been variably tested using different reporter constructs (Beyer et al. 2021; Brenner et al. 1994; Casper, Jones, and McCarthy 2007; Casper and McCarthy 2006; Garcia et al. 2004; Gregorian et al. 2009; Herrmann et al. 2008; Mori et al. 2006; Park et al. 2018; Slezak et al. 2007; Srinivasan et al. 2016; Tanaka et al. 2008; Winchenbach et al. 2016; Yu, Nagai, and Khakh 2020; Zhou et al. 2022).

Aldh1l1‐Cre/ERT2 lines have been increasingly deployed given their high astrocyte specificity, broad coverage, and strong levels of loxP recombination in most adult areas of the CNS under healthy and disease‐related conditions (Hu et al. 2020; Sadick et al. 2022; Srinivasan et al. 2016; Winchenbach et al. 2016). In neuroinflammatory conditions, such as experimental autoimmune encephalomyelitis (EAE), a widely studied preclinical model of CNS autoimmune demyelinating disease, peripheral immune cells infiltrate into the CNS from the peripheral lymph nodes and spleen (Reich, Lucchinetti, and Calabresi 2018), and the latter organ is noted to exhibit Aldh1l1‐Cre/ER2‐mediated recombination (Nemeth et al. 2023). Crosstalk between peripheral immune cells and resident CNS cells, including vascular endothelial cells, pericytes, astrocytes, microglia, neurons, and oligodendrocytes, contributes to lesional pathogenesis (Absinta et al. 2021; Benallegue et al. 2021; Efremova et al. 2020; Guttenplan et al. 2021; Han et al. 2021; Ito et al. 2019; Kiss et al. 2023; Liddelow et al. 2017; Linnerbauer, Wheeler, and Quintana 2020; Sanmarco, Polonio et al. 2021; Williams et al. 2020).

We report here that Aldh1l1‐Cre/ERT2‐mediated recombination in *Tg(Aldh1l1‐cre/ERT2)1Khakh* mice occurs in CNS infiltrating immune cells during the proinflammatory peak of EAE. We performed flow cytometry in the spleen and spinal cord of both Aldh1l1‐Cre (*Tg(Aldh1l1‐cre/ERT2)1Khakh*) and Gfap‐Cre (*Tg(Gfap‐cre)73.12Mvs*)‐driven reporter (*Gt(ROSA)26Sor*) mice to characterize which immune cell populations undergo recombination under healthy conditions and EAE. We found that mGfap‐Cre‐driven recombination did not lead to *lox*P recombination in peripheral immune cells during peak EAE.

Therefore, the design of functional genetic modulation studies targeting astrocytes must account for possible untargeted Aldh1l1‐Cre/ERT2‐driven effects in the peripheral immune system, which may additionally contribute to mutant phenotypes.

## 2 Materials and Methods

### 2.1 Antibodies

Initial flow cytometry screening experiments measuring the expression of tdTomato in CD45^+^ cells were performed using anti‐CD45 PE/Cy7 (Biolegend, Clone 30‐F11, cat. 103113 [1:100] with live/dead staining using Zombie Yellow (Biolegend, cat. 423103 [1:200]). Subsequent experiments measuring surface markers for multiple immune cell populations used a cocktail of antibodies outlined in Table S1. Primary antibodies used for immunohistochemistry (IHC) were as follows: CD45, 1:200, host rat, BD Pharmigen 550539; Gfap, 1:200, host mouse, ThermoFisher 14‐9892‐82; tdTomato/RFP, 1:500, host rabbit, Rockland 600‐401‐379.

### 2.2 Mice

Astrocyte‐targeted reporter mouse lines were generated by crossing the following mouse lines: Two astrocyte‐targeted promoter‐driven Cre‐recombinase lines (*Tg(Aldh1l1‐Cre/ERT2)1Khakh* and *Tg(Gfap‐Cre)73.12Mvs*) were crossed to the *Gt(ROSA)26Sor* line, which contains a tdTomato sequence preceded by a *loxP*‐flanked STOP cassette. *Aldh1l1‐Cre/ERT2* (B6N.FVB‐Tg(Aldh1l1‐Cre/ERT2)1Khakh/J) mice and *mGfap‐Cre* (B6.Cg‐Tg(Gfap‐Cre)73.12Mvs/J) mice were genetically engineered in the laboratories of Baljit Khakh (UCLA) and Michael Sofroniew (UCLA), respectively, and are commercially available from Jackson Laboratories (Strain #012886 and #031008, respectively). The *Gt(ROSA)26Sor* mice were genetically engineered in the laboratory of Philippe Soriano (Icahn School of Medicine at Mount Sinai) (Sadick et al. 2022; Soriano 1999) and are commercially available from Jackson Laboratories (Strain #003474). *Aldh1l1‐Cre/ERT2:ROSA* and *Gfap‐Cre*:*ROSA* animals express tdTomato within the cell populations expressing the respective promoters. Activation of Aldh1l1‐cre/ERT2 was performed 2 weeks prior to EAE induction using five daily intraperitoneal injections of 100 mg/kg tamoxifen (20 mg/mL solution dissolved in corn oil). Gfap‐Cre activity is constitutive and did not require activation. Mice used in all groups were a combination of males and females. Transgenic mice were homozygous for Rosa and heterozygous for Cre (as breeding parents consisted of a Cre‐positive female and Cre‐negative male). Genotyping primers are outlined in Table S2. Polymerase chain reaction (PCR) conditions for all primer sets were performed per Jackson Laboratories’ instructions.

### 2.3 EAE

EAE was induced at 8–10 postnatal weeks. Mice were anesthetized with 2%–3% isoflurane in O_2_ and then maintained under 1.5% while subcutaneously injected at the lumbar midline with 1 cm^3^ of 1:1 MOG_35–55_ (MEVGWYRSPFSRVVHLRNGK‐COOH (Stanford PanPeptide); 15 mg/mL solution in 1× PBS) to complete Freund's adjuvant (CFA) (5 mg desiccated *Mycobacterium tuberculosis* (BD cat. 231141) per 1 mL of incomplete Freund's adjuvant (IFA; ThermoScientific cat. 77145)), followed by intraperitoneal injection of 60 ng pertussis toxin (0.1 cm^3^ of 0.6 mg/mL in 1× PBS; Hooke laboratories cat. BT‐0105) on Day 0 (1–5 h after MOG_35–55_) and Day 2 (47–60 h after MOG_35–55_). Healthy controls (HCs) received CFA emulsion injections with no MOG_35–55_ followed by pertussis toxin injections, as above. Mice were rated daily on a standard 5‐point motor scale starting at 7 days from induction: 0, no symptoms; (1) floppy tail; (2) hind limb weakness (paraparesis); (3) hind limb paralysis (paraplegia); (4) forelimb and hind limb paralysis; and (5) death. Spleen and spinal cords were harvested at variable time points (including 4 and 28 days from disease onset, as noted below) and processed for flow cytometry.

### 2.4 Flow Cytometry

**FIGURE 1:**
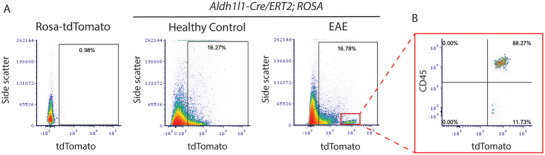
***Aldh1l1‐Cre/ERT2;ROSA* mice with EAE are characterized by the presence of CD45^+^/tdTomato^+^ cells in the inflamed spinal cord**. Spinal cords from mice with EAE and under healthy conditions were harvested 6 weeks after treatment with tamoxifen (at 28 days post EAE immunization), homogenized and centrifuged in a 70%/30% Percoll gradient to enrich the astrocytic cell population. DNA recombination was assessed following the expression of the tdTomato reporter gene in flow cytometry. (A) As expected, no tdTomato^+^ cells were observed in *ROSA* control mice (with no *Aldh1l1‐Cre/ERT2*). tdTomato^+^ cells were detected in *Aldh1l1‐Cre/ERT2;ROSA* mice, indicating successful DNA recombination. *Aldh1l1‐Cre/ERT2; ROSA* mice with EAE were characterized by the presence of an additional distinctive population of cells positive for tdTomato (red rectangle) which was absent in *Aldh1l1‐Cre/ERT2;ROSA* healthy controls. (B) Flow cytometry analysis revealed that this distinct tdTomato^+^ cell population was positive for the leukocyte common antigen, CD45. EAE, experimental autoimmune encephalomyelitis.

**FIGURE 2:**
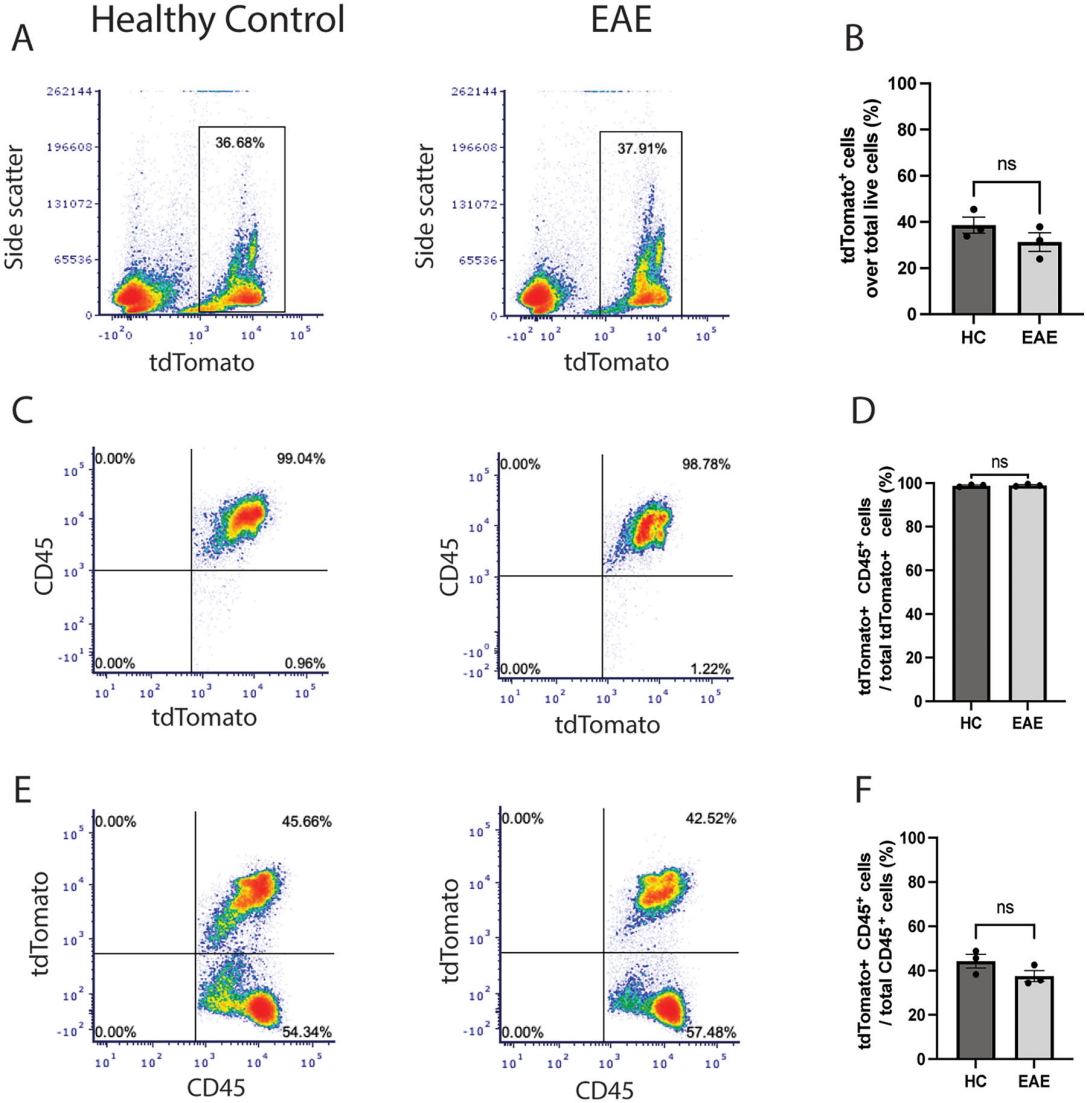
**Aldh1l1‐Cre/ERT2 drives *loxP* recombination in CD45^+^ leukocytes**. Spleens were harvested from *Aldh1l1‐Cre/ERT2;ROSA* mice with EAE and from healthy controls (HC) 6 weeks after treatment with tamoxifen. DNA recombination was assessed following the expression of the tdTomato reporter gene in flow cytometry. (A and B) Expression of the tdTomato reporter gene was observed in the spleen of both HC (range: 33.8%–45.4% of total live cells) and EAE (range: 31.3%–37.9%) mice. The proportion of cells positive for tdTomato over the total number of live cells was not significantly different between HC and EAE mice. (C and D) Almost all tdTomato^+^ splenocytes were CD45^+^, indicating that the Aldh1l1 promoter drives *loxP* recombination in leukocytes. Mice with EAE and HCs showed similar proportions of tdTomato^+^ CD45^+^ cells over the total number of tdTomato^+^ cells (HC, mean = 98.2; EAE, mean = 98.9). (E and F) tdTomato^+^ cells represented about 40% of the total live CD45^+^ cells in the spleen in both HC (range: 44.3%–48.8%) and EAE (range: 34.4%–42.5%) mice. No statistically significant differences were observed between the two groups. (B, D, and F) *n* = 3 per group; unpaired Student's *t* test. EAE, experimental autoimmune encephalomyelitis.

**FIGURE 3:**
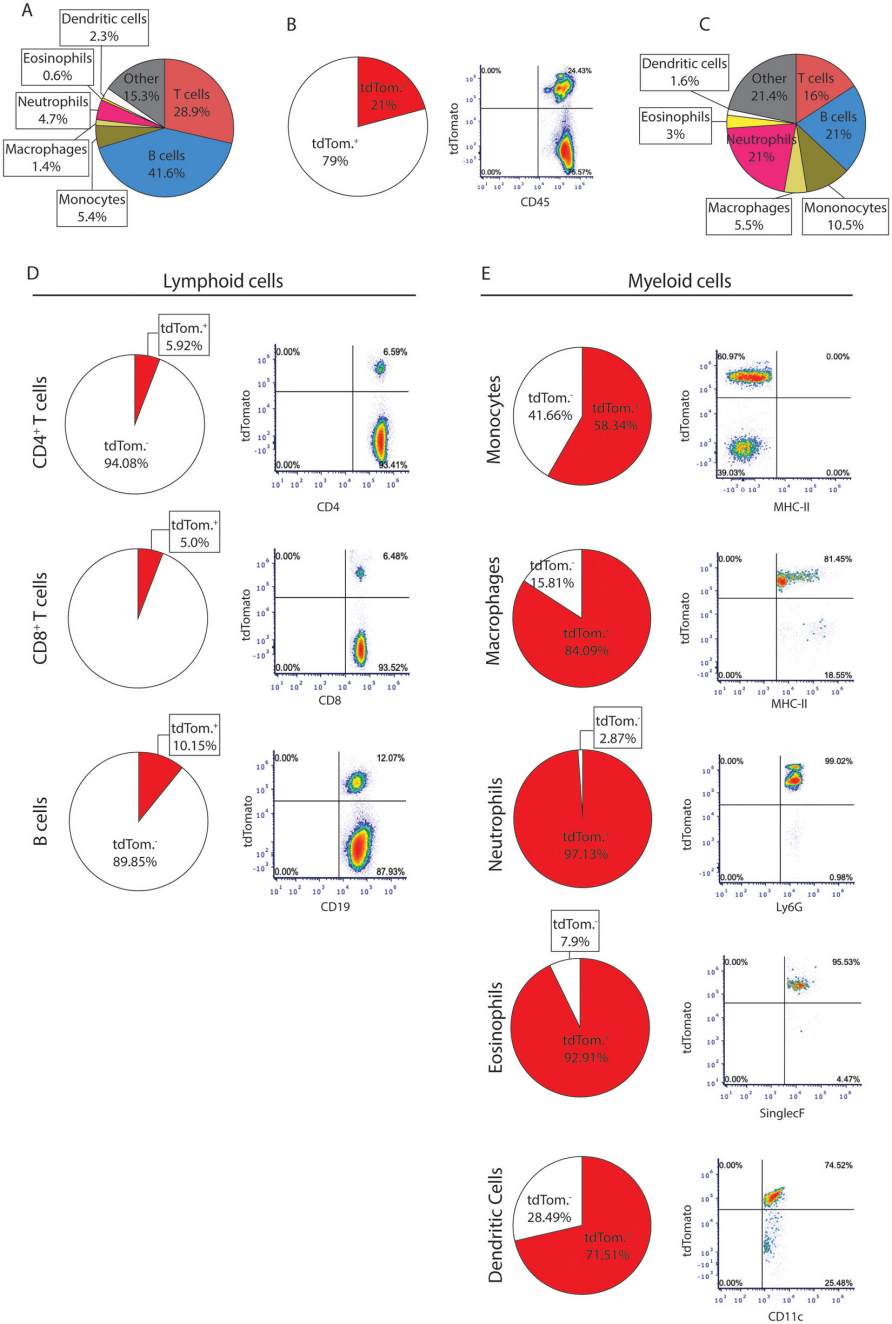
**Characterization of CD45^+^ tdTomato^+^ cells in *Aldh1l1‐Cre/ERT2* mice**. Flow cytometry of the CD45^+^ tdTomato^+^ cells from the spleen of *Aldh1l1‐Cre/ERT2* mice 3 weeks after tamoxifen treatment. Pie charts show averaged cell populations’ frequency (*n* = 3 mice), whereas density plots show representative FACS analysis per each cell population. (A) Pie chart shows the relative frequency of the analyzed leukocyte cell types over the total number of CD45^+^ cells in spleen. The antibody panel used in this experiment did not resolve 15.3% of the CD45^+^ cells. (B) On average, 21% of CD45^+^ cells were tdTomato^+^. (C) Pie charts show the proportion of each leukocyte subset over the total number of CD45^+^ tdTomato^+^ cells. (D and E) Pie charts show the average proportion of tdTomato^+^ cells in each specific cell type from the lymphoid (D) and myeloid (E) lineage. Representative density plots show the frequency of tdTomato^+^ cells within each leukocyte subtype. Gating strategies are depicted in Figures S1 and S2.

**FIGURE 4:**
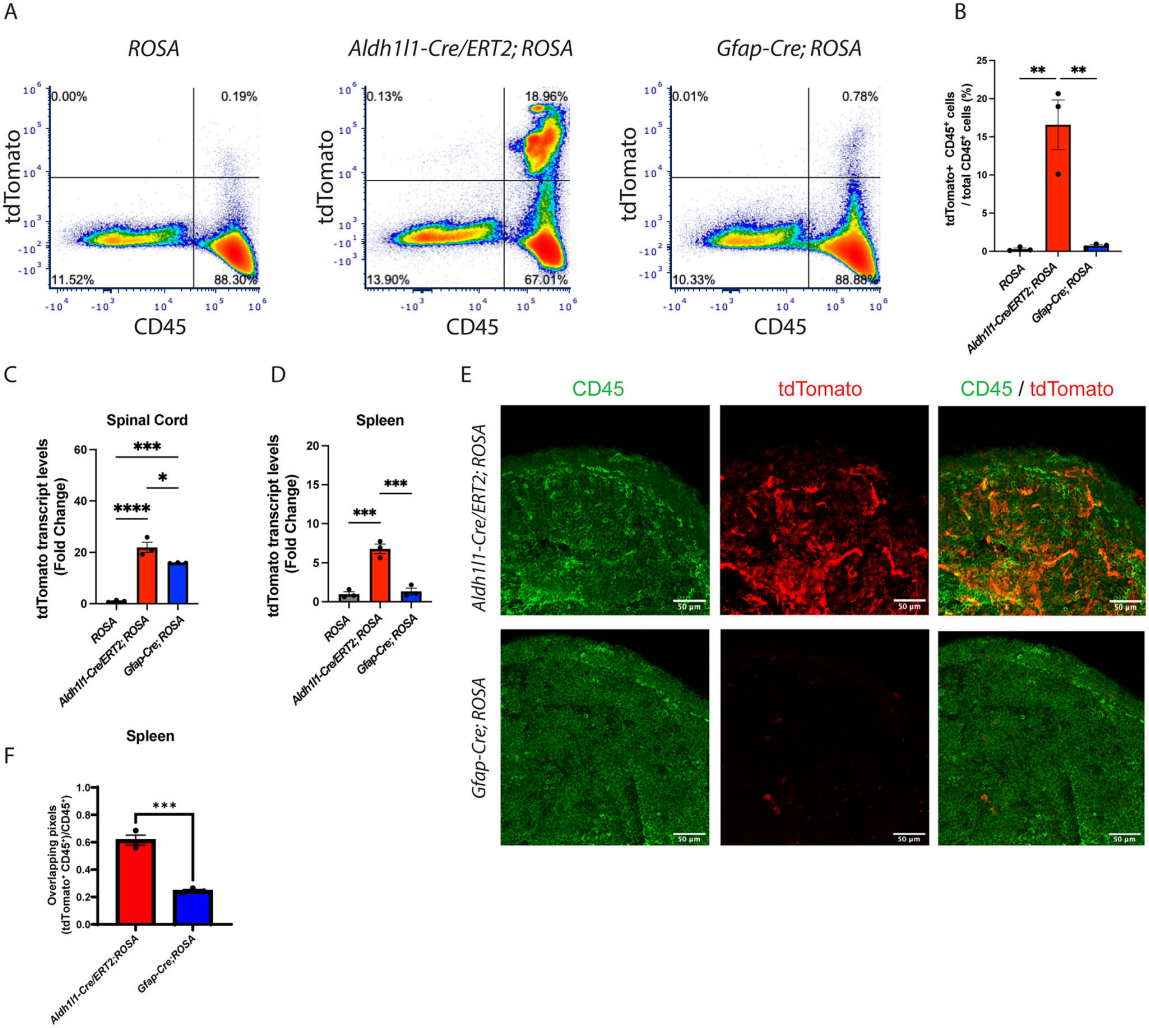
**Gfap‐Cre does not significantly drive *loxP* recombination in splenic leukocytes**. tdTomato reporter expression in *Aldh1l1‐Cre/ERT2;ROSA* 3 weeks after treatment with tamoxifen and untreated *Gfap‐Cre;ROSA* mice (A–D). *ROSA* mice (with no Cre) were used as controls. (A and B) Flow cytometry (representative density plots in A; quantification in B) analysis shows a significant greater proportion of tdTomato^+^ CD45^+^/CD45^+^ leukocytes in the spleen of *Aldh1l1‐Cre/ERT2;ROSA* (mean 19.4) compared with *Gfap‐Cre;ROSA* (mean 2.5) and control mice (mean 0.94), in which tdTomato^+^ cells were nearly undetectable, *Aldh1l1‐Cre/ERT2;ROSA* versus *Gfap‐Cre;ROSA p* < 0.005, *Aldh1l1‐Cre/ERT2;ROSA* versus *ROSA*, *p* < 0.005, *Gfap‐Cre;ROSA* versus *ROSA*, *p* = 0.83; *n* = 3 per group. One‐way ANOVA with Tukey's multiple comparison test. (C and D) RT‐PCR analysis of tdTomato transcript levels revealed a significant upregulation in the spinal cord of both *Aldh1l1‐Cre/ERT2;ROSA* and *Gfap‐Cre;ROSA* compared with controls (C) (means 21.98, 15.92, and 1.0, respectively; *Aldh1l1‐Cre/ERT2;ROSA* vs. *Gfap‐Cre;ROSA*, *p* < 0.05, *Aldh1l1‐Cre/ERT2;ROSA* vs. *ROSA*, *p* < 0.0001, *Gfap‐Cre;ROSA* vs. *ROSA*, *p* < 0.0005). *n* = 3 per group. One‐way ANOVA with Tukey's multiple comparison test. tdTomato transcript levels were significantly increased in the spleen of *Aldh1l1‐Cre/ERT2;ROSA* mice compared with *Gfap‐Cre;ROSA* mice and *ROSA* controls (D) (means 6.79, 1.33, and 1.0, respectively; *Aldh1l1‐Cre/ERT2;ROSA* vs. *Gfap‐Cre;ROSA*, *p* < 0.0005, *Aldh1l1‐Cre/ERT2;ROSA* vs. *ROSA*, *p* < 0.0005, *Gfap‐Cre;ROSA* vs. *ROSA*, *p* = 0.87), suggesting non‐astrocyte specific *loxP* recombination in *Aldh1l1‐Cre/ERT2* mice but not in *Gfap‐Cre* mice. *n* = 3 per group. One‐way ANOVA with Tukey's multiple comparison test. (E and F) Immunohistochemistry images (E) and co‐localization analysis (F) of tdTomato and CD45 staining in spleens of healthy, tamoxifen‐treated *Aldh1l1‐Cre/ERT2;ROSA* and *Gfap‐Cre;ROSA* mice confirmed the presence and significantly increased levels of co‐localizing tdTomato^+^ (red) and CD45^+^ (green) signal in *Aldh1l1‐Cre/ERT2;ROSA* mice compared to *Gfap‐Cre;ROSA* mice (mean 0.62 vs. 0.24, respectively, *p* < 0.001). Samples from both groups were harvested 2 weeks post‐tamoxifen treatment. Scale bar 50 µm, *n* = 3 per group. Unpaired Student's *t*‐test.

**FIGURE 5:**
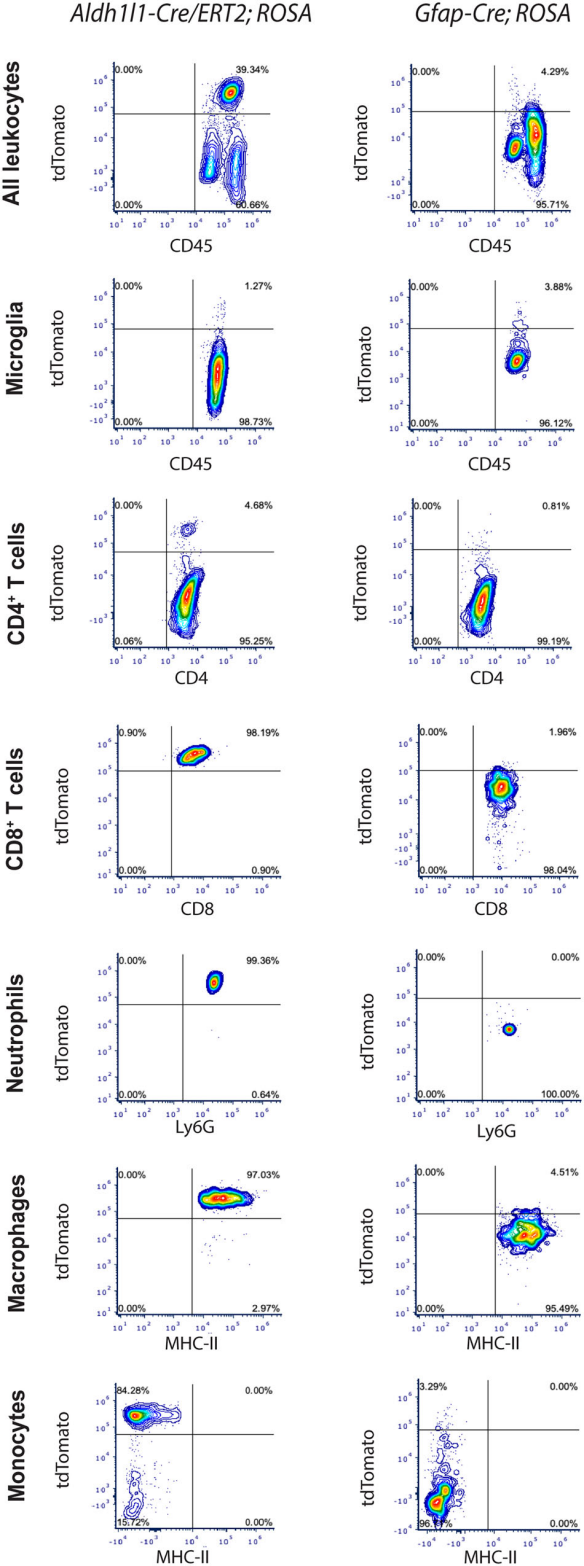
**Representative FACS density plots for tdTomato+ leukocytes from EAE spinal cords of *Aldh1l1‐Cre/ERT2;ROSA* and *Gfap‐Cre;ROSA* mice at peak disease**. *Aldh1l1‐Cre/ERT2;ROSA* mice show tdTomato^+^ populations of neutrophils, macrophages, monocytes, and CD4^+^ T cells, whereas *Gfap‐Cre;ROSA* mice lack these populations. Gating strategies are depicted in Figures S3 and S4.

Mice were anesthetized and perfused with 20 mL ice‐cold 1× PBS. Spinal cords and spleens were collected in cold PBS and mechanically dissociated. Spleen samples were passed through a 70‐µm filter, centrifuged, and then incubated in 3 mL of 1× red blood cell (RBC) lysis buffer (BioLegend) for 5 min at 4°C, washed with 1× PBS, centrifuged, and then resuspended in 10 mL RPMI. Spinal cords were homogenized in a tissue grinder in 6 mL of cold RPMI, centrifuged, and then resuspended in 5 mL of 70% Percoll. Cells were separated from myelin using a 70%/30% Percoll gradient in centrifugation at 800*g* for 20 min at 25°C. Cells were collected from the Percoll interface and passed through a 70‐µm filter, rinsed in RPMI, centrifuged, and resuspended in 1 mL RPMI. Cell suspensions were counted. One million and 4 to 5 million cells for spleen and spinal cord, respectively, were subjected first to a Zombie Yellow stain (Biolegend, 423103) and wash, followed by the addition of FC block. Cell samples were then incubated with cell surface marker antibodies (as listed in the Antibodies section) for 20 min at 4°C followed by fixation with Fixation Medium A (Life Technologies, GAS001S5). Samples were then run on a Cytek Aurora Flow Cytometer. Flow cytometry data were analyzed and represented for figures using either FCS Express software (De Novo) (Figures 1, 2, 3, 4, 5; Figures S1 and S2) or FloJo software (BD Bioscience) (Figures S3–S6) at the Flow Cytometry CoRE at Mount Sinai. Flow cytometry for spinal cord tissues was set to run up to 10,000 events over live cells. Forward and side scatter were used to gate cells excluding debris and cell aggregates, and Zombie Yellow was used to exclude dead cells. Remaining splenic cells were then gated as outlined in Figure S1 to measure leukocytes of the lymphoid lineage using CD45, CD3, CD4, CD8, and CD19 markers and in Figure S2 to measure leukocytes of the myeloid lineage using CD45, CD11b, Siglec‐F, Ly6C, Ly6G, F4/80, and MHC‐II. Spinal cord cells were gated as outlined in Figures S3 and S4 to measure leukocytes of the lymphoid and myeloid lineages, respectively.

**TABLE 1:** Proportion of tdTomato^+^ leukocytes from the spinal cord of *Aldh1l1‐Cre/ERT2;ROSA* (A) and *Gfap‐Cre;ROSA* (G) mice with EAE.

		Proportions of tdTomato^+^ cells (%)		
Cell type	Marker(s)	*Aldh1l1‐Cre* (A) (mean ± SEM)	*Gfap‐Cre* (G) (mean ± SEM)	*p* value
All leukocytes	CD45^+^	36.89 ± 4.31	4.51 ± 0.86	A > G; *p = 0.0251
Microglia	CD11b^+^CD45^low^	1.63 ± 0.28	3.40 ± 0.82	*p *= 0.0887
CD4 T cells	CD3^+^CD4^+^	3.81 ± 0.58	1.03 ± 0.46	A > G; *p = 0.0096
CD8 T cells	CD3^+^CD8^+^	98.90 ± 0.50	1.95 ± 0.29^a^	A > G; ****p < 0.0001
B cells	CD19^+^	N/D	N/D	N/A
Neutrophils	CD11b^+^Ly6G^+^	99.63 ± 0.15	1.46 ± 1.46	A > G; ****p < 0.0001
Macrophages	CD11b^+^CD45^high^Ly6C^+^F4/80^+^ MHC‐II^+^	95.74 ± 1.20	5.21 ± 1.01	A > G; ****p < 0.0001
Monocytes	CD11b^+^CD45^high^Ly6C^+^MHC‐II^−^	84.71 ± 2.71	5.62 ± 1.03	A > G; ****p < 0.0001

*Note*: Spinal cords were harvested for flow cytometry analysis at Day 4 after the onset of neurological disease. Statistical differences were assessed with Student's *t* test (*p* < 0.05). *n* = 4 per group, with an exception for the analysis of CD8^+^ T cells as this population was detected in only two out of four mice within the Gfap‐Cre group (marked with a hashtag in the table).

Abbreviations: N/D, not detected; N/A, not applicable.

^a^ *n* = 2 for the group as CD8^+^ T cells were detected in only two out of the four mice, *n* = 4 per group for all other analyses.

*p < 0.05; ****p < 0.0005.

### 2.5 Real‐Time PCR (RT‐PCR)

Healthy mice were anesthetized and perfused with 20 mL ice‐cold 1× PBS. Spinal cords and spleens were harvested and snap frozen on dry ice prior to RNA extraction. Briefly, tissue was homogenized in 1 mL of TRIzol using a glass homogenizer at 900 rpm. Samples were then incubated at room temperature for 5 min. Following, 200 µL of chloroform was added, and the tube was shaken vigorously for 15 s, incubated for 3 min at room temperature, and centrifuged at maximum speed for 15 min at 4°C. The upper aqueous phase (∼350 µL) was carefully transferred to a new 1.5 mL tube using a P200 tip, avoiding the interphase. Finally, an equal volume of isopropanol (∼350 µL) was added to the aqueous phase and mixed thoroughly by pipetting. RNA was eluted using the RNeasy Mini Kit (Qiagen, #74104) following manufacturers’ instructions, and cDNA was synthesized with a cDNA Super Mix (Quanta Biosciences #95048‐100). RT‐PCRs were performed using a thermocycler (ABI7900HT; Applied Biosystems). The PCR conditions used were as follows: 95°C for 2 min, 40 cycles of 95°C for 15 s, 55°C for 15 s, and 72°C for 30 s. Primer sequences for tdTomato were as follows: F‐5′‐ATCGTGGAACAGTACGAGCG‐3′ and R‐5′‐TGAACTCTTTGATGACGGCCA‐3′, as used in previously published work (Johnson et al. 2018), and for the housekeeping genes β‐actin (Actb), Gapdh and α‐tubulin were as follows: β‐actin: F‐5′‐AGGTGACAGCATTGCTTCTC‐3′ and R‐5′‐GCTGCCTCAACACCTCAAC‐3′, Gapdh: F‐5′‐CTTGCTCAGTGTCCTTGCTG‐3′ and R‐5′‐TGCGACTTCAACAGCAACTC‐3′, and α‐tubulin: F‐5′‐CTGGAGCAGTTTGACGACAC‐3′ and R‐5′‐TGCCTTTGTGCACTGGTATG‐3′. mRNA measurements were normalized using a robust global normalization algorithm. All control crossing threshold (Ct) values were corrected by the median difference in all samples from Actb. All samples were then normalized by the difference from the median Ct of the three corrected control gene Ct levels in each sample, with the value converted to a nominal copy number per cell by assuming 2500 Actb mRNA molecules per cell and an amplification efficiency of 93% for all reactions.

### 2.6 IHC

Spleens and spinal cords were dissected from animals perfused with 10 mL ice‐cold 1× PBS followed by 10 mL 4% PFA‐1× PBS, tissues were subjected to 2 h postfixation in 4% PFA‐1× PBS followed by storage in 30% sucrose‐1× PBS at 4°C until sectioning. Immunostaining was performed on 25 µm axial sections. For all antibody staining, sections underwent antigen retrieval in citrate (pH 6.0; 100°C) for 20 min. For CD45, sections were treated with 0.5 mg/mL protease XIV (Sigma‐Aldrich) at 37°C for 5 min, then blocked and incubated with primary antibodies overnight at 4°C (antibodies and concentrations listed above), followed by 3× PBST wash, a 1 h incubation with Alexa Fluor secondary antibodies at 1:500 (ThermoFisher), 3× PBST wash and mount with DAPI. Samples were examined using a Leica Microsystems confocal microscope, and stacks were collected with z of 1 µm.

### 2.7 Morphometric Analysis

Morphometric analyses were performed using the NIH ImageJ and Leica LAS software, and all analyses were performed blinded to treatment group and genotype. For colocalization analyses in vivo (tdTomato and CD45, tdTomato and Gfap), immunohistochemical stains were analyzed with the ImageJ Just Another Colocalization Plugin (JACoP) through each plane of the z‐stack in at least two (range: 2–3) spleen and lumbar spinal cord cross sections per mouse.

### 2.8 Statistics

Results are reported as mean ± SEM. Student's *t* test and Mann–Whitney *U* tests were used to compare two groups of unmatched samples. One‐way ANOVA and Kruskal–Wallis *H* tests were used to compare more than two groups with multiple comparisons. In all cases, *p* less than 0.05 was considered significant.

## 3 Results

### 3.1 CD45^+^ Cells Within the Spinal Cord Exhibit *Aldh1l1‐Cre/ERT2*‐Driven loxP Recombination During EAE

*Aldh1l1‐Cre/ERT2;ROSA* mice were generated in our laboratory to enable live multiphoton imaging of immune cell trafficking through the glia limitans during EAE. To validate loxP recombination in this model, flow cytometry was performed on spinal cord samples from healthy and EAE animals. To our surprise, two distinct cell populations expressing tdTomato in EAE were detected (Figure 1A), one of which was positive for CD45, a cell surface marker identifying leukocytes (Figure 1B).

### 3.2 A Wide Range of CD45^+^ Cell Subtypes Within the Spleen Exhibit *Aldh1l1‐Cre/ERT2*‐Driven loxP Recombination Under Healthy Conditions and During EAE

To test whether the CD45^+^ tdTomato^+^ cells in *Aldh1l1‐Cre/ERT2;ROSA* mice originate from the periphery, spleens were harvested from HCs and EAE mice at 6 weeks after tamoxifen treatment (which coincided with 28 days after EAE induction in EAE mice). A distinctive tdTomato^+^ cell population was seen in HCs and EAE mice in similar proportions (Figure 2A,B), and nearly all cells in this population were CD45^+^ in both groups (Figure 2C,D). Of all CD45^+^ splenic cells, roughly 40% of these cells expressed tdTomato in both groups (Figure 2E,F), suggesting that Aldh1l1‐Cre/ERT2 is active in a subset of immune cells within the spleen, as previously reported (Nemeth et al. 2023). Control experiments were performed measuring tdTomato expression in splenic CD45^+^ cells of healthy tamoxifen‐injected *Gfap‐Cre;ROSA* mice and healthy, non‐tamoxifen‐injected *Aldh1l1‐Cre/ERT2; ROSA* mice, both of whom showed minimal numbers of these cells compared to healthy tamoxifen‐injected *Aldh1l1‐Cre/ERT2; ROSA* mice (Figure S5). Therefore, tamoxifen itself was not responsible for off‐target flox recombination and was required to induce high levels of loxP recombination in splenic CD45^+^ cells of *Aldh1l1‐Cre/ERT2; ROSA* mice.

To determine which immune cell subtypes in the spleen are involved, multidimensional flow cytometry was performed with both lymphoid (CD3, CD4, CD8, and CD19) and myeloid cell markers (CD11b, Siglec‐F, Ly6C, Ly6G, F4/80, and MHC‐II) to measure subtypes of CD45^+^ tdTomato^+^ cells from HC spleens at 3 weeks after tamoxifen treatment (*n* = 3). Respective gating strategies for lymphoid and myeloid cells are outlined in Figures S1–S4. Within the total splenic CD45^+^ cell population, average proportions of neutrophils (4.7%), dendritic cells (2.3%), monocytes (5.4%), macrophages (1.4%), eosinophils (0.6%), T cells (28.9%), and B cells (41.6%) were comparable to previous reports (https://www.miltenyibiotec.com/US‐en/resources/macs‐handbook/mouse‐cells‐and‐organs/mouse‐cell‐sources/spleen‐mouse.html; https://assets.thermofisher.com/TFS‐Assets/LSG/brochures/I‐076357%20cell%20count%20table%20topp_WEB.pdf) (Figure 3A). Of all CD45^+^ cells, 21% expressed tdTomato (Figure 3B) and of these cells, the average distribution of subtypes was as follows: neutrophils (21%), dendritic cells (1.6%), monocytes (10.5%), macrophages (5.5%), eosinophils (3%), T cells (16%), and B cells (21%) (Figure 3C). Although lymphoid cells (T and B) represented the highest proportion of all tdTomato positive cells in the spleen, as they are the most abundant leukocyte types, within their respective classes, only a small percentage of lymphoid cells undergo flox mediated recombination (an average of 5.92% CD4^+^ T cells, 5% CD8^+^ T cells, and 10.15% B cells (Figure 3D)) compared to the majority of myeloid cells (58.34% monocytes, 84.09% macrophages, 97.13% neutrophils, 92.91% eosinophils, and 71.51% dendritic cells (Figure 3E)).

### 3.3 *Gfap‐Cre‐*Driven loxP Recombination Occurs in the Spinal Cord but Not in the Spleen

To test whether the widely used Gfap‐Cre recombinase also drives recombination in leukocytes, spleens were harvested for flow cytometry from *Gfap‐Cre;ROSA*, and from *Aldh1l1‐Cre/ERT2;ROSA* and *ROSA* mice for comparison. Although *Aldh1l1‐Cre/ERT2;ROSA* mice showed a population of tdTomato^+^ CD45^+^ cells*, Gfap‐Cre;ROSA* and *ROSA* mice did not (Figure 4A,B). Under healthy conditions, RT‐PCR for *tdTomato* showed the presence of transcript in the spinal cord of both *Aldh1l1‐Cre/ERT2;ROSA* and *Gfap‐Cre;ROSA* mice (Figure 4C), whereas in the spleen, only *Aldh1l1‐Cre/ERT2;ROSA* demonstrated *tdTomato* transcript (Figure 4D). IHC was performed to validate the increased presence of tdTomato‐expressing CD45^+^ cells in healthy tamoxifen‐treated *Aldh1l1‐Cre/ERT2;ROSA* spleens compared to healthy tamoxifen‐treated *Gfap‐Cre;ROSA* spleens (Figure 4E,F). The histology showed clear and robust tdTomato^+^ signal in spleens from *Aldh1l1‐Cre/ERT2;ROSA* mice, whereas this signal was minimally seen in the spleens from *Gfap‐Cre;ROSA* mice (Figure 4E). Colocalization analysis between tdTomato and CD45 immunoreactivity revealed a significantly greater overlap between these two markers in spleens from *Aldh1l1‐Cre/ERT2;ROSA* mice compared with *Gfap‐Cre;ROSA* mice (Figure 4F). Spinal cords from these mice showed tdTomato expression in Gfap‐positive cells (i.e., astrocytes). The analysis showed tdTomato expression in Gfap^+^ cells in both *Aldh1l1‐CreERT2;ROSA* and *Gfap‐Cre;ROSA* mice (data not shown), as expected and consistent with previously published work (Amatruda et al. 2022; Garcia et al. 2004; Horng et al. 2017; Kiss et al. 2023; McAlpine et al. 2021; Srinivasan et al. 2016).

### 3.4 Aldh1l1‐Cre/ERT2, but Not Gfap‐Cre, Drives tdTomato Expression in Multiple Infiltrating Immune Cell Populations in the Spinal Cord During EAE

In spinal cords harvested at peak EAE (4 days from disease onset), *Aldh1l1‐Cre/ERT2;Rosa‐tdTomato* mice demonstrated in comparison to *Gfap‐Cre;Rosa‐tdTomato* mice significant increased proportions of tdTomato^+^ CD45^+^ cells (mean 36.9 vs. 4.5, *p* = 0.02), CD4^+^ T cells (mean 3.8 vs. 1.0, *p* = 0.0096), CD8^+^ T cells (mean 98.9 vs. 1.95, *p* < 0.0001), neutrophils (99.6 vs. 1.6, *p* < 0.0001), macrophages (95.7 vs. 5.2, *p* < 0.0001), and monocytes (84.7 vs. 5.6, *p *< 0.0001) (Figure 5) (Table 1).

## 4 Discussion

Transgenic mouse lines using the Cre‐loxP system remain a critical tool for investigating the role of astrocyte signaling in CNS neuroinflammatory and neurodegenerative disease processes (Slezak et al. 2007; Srinivasan et al. 2016; Yu, Nagai, and Khakh 2020). We report here that Aldh1l1‐Cre/ERT2, but not Gfap‐Cre, leads to recombination in a wide range of peripheral and CNS infiltrating immune cells under healthy and neuroinflammatory conditions. Our findings are consistent with recent histologic measurements demonstrating tdTomato reporter expression in the spleen (and exocrine glands) of Aldh1l1‐Cre/ERT2 mice (Nemeth et al. 2023). Here, we found further that Aldh1l1‐Cre/ERT2 leads to off‐target flox‐mediated recombination in a large proportion of immune cells, including dendritic cells, neutrophils, monocytes, macrophages, CD4^+^ and CD8^+^ T cells, and B cells. In neuroinflammatory conditions, these effects may contribute to CNS phenotypes otherwise attributed exclusively to astrocytes.

An advantage of Aldh1l1‐Cre/ERT2 lies in its robust levels of recombination and greater coverage of CNS astrocytes over older Gfap‐Cre recombinases (Hu et al. 2020; Srinivasan et al. 2016; Winchenbach et al. 2016). In single‐cell RNA sequencing of mouse cortex and hippocampus, ACSA‐2 antibody‐recovered cells showed enrichment of Gfap in most but not all clusters of astrocytes (Batiuk et al. 2020). In human brain histological studies of astrocyte‐specific markers, whereas nearly all (99%) Gfap‐expressing cells expressed Aldh1l1, only 87% of Aldh1l1‐expressing cells expressed Gfap (Forrest et al. 2023). Quantitative assessment of the inducible line, GFAP‐Cre/ERT2 *(Tg(GFAP‐Cre/ERT2)13Kdmc)*, demonstrated high (> 80%) specificity in cortex, striatum, hippocampus, and cerebellar granule layers and high (74%) coverage in cortex but low‐to‐moderate (50%–60%) coverage in these remaining areas (Park et al. 2018). Therefore, the decision to use Gfap‐Cre over Aldh1l1‐Cre may sacrifice coverage to increased specificity in the neuroinflammatory context. As our understanding of astrocyte heterogeneity evolves, new subtype‐specific promoter‐driven Cre‐recombinases may optimize specificity in astrocyte subpopulations of interest.

Our finding that high proportions of myeloid immune cells and lower proportions of lymphoid immune cells undergo Aldh1l1‐Cre‐mediated recombination raises the possibility that Aldh1l1 contributes to the identity, differentiation, and/or effector pathways of innate immunity. Shared epigenetic and effector pathways between innate immune cells and astrocytes represent an interesting area of inquiry, suggesting conserved mechanisms of inflammatory disease within and outside the CNS.

Intriguingly, among the immune cell subtypes, CD8^+^ T cells showed a marked difference in the proportion of tdTomato^+^ cells in the spleen (5%) compared to the spinal cord (98.9%). One possibility is that an Aldh1l1‐expressing functional subgroup of CD8^+^ T cells from the spleen and lymph nodes selectively infiltrates into the spinal cord during EAE. Alternatively, Aldh1l1 may be induced selectively in CNS‐infiltrated CD8^+^ T cells by local factors during EAE. Regulatory CD8^+^ T cells play a critical role in driving remission in EAE and other autoimmune diseases (Li et al. 2022; Saligrama et al. 2019), so the relationship between Aldh1l1 activity and regulatory function within the CNS during autoinflammation is an interesting potential interaction to explore in future work.

A limitation of this study includes our testing of a constitutive Gfap‐Cre line that is widely used, commercially available, and was available to our group, instead of an inducible Gfap‐Cre line. Sparing of the peripheral immune system should be confirmed in Gfap‐Cre/ERT2 lines prior to their use. Another caveat is the possibility that tamoxifen, as used in the Aldh1l1‐Cre/ERT2 line, could itself lead to off‐target flox recombination in immune cells, though this was deemed less likely given the low numbers seen in tamoxifen‐treated *Gfap‐Cre;ROSA* controls (Figure S5) as well as previously published work using tamoxifen in other inducible *Gfap‐Cre* lines (Casper, Jones, and McCarthy 2007; Park et al. 2018).

## 5 Conclusion

Flox‐mediated recombination driven by the Aldh1l1‐cre/ERT2, but not Gfap‐cre, recombinase occurs in multiple peripheral immune cell populations in addition to astrocytes. The use of *Gfap‐cre* lines may provide a more specific means to targeting astrocytes in disease models involving immune cell infiltration into the CNS. Gain and loss of function experiments using the Aldh1l1‐Cre/ERT2 recombinase in models of neuroinflammatory, ischemic, and neurodegenerative diseases must account for potential mutational contributions in both astrocytes and peripheral immune cells to reported phenotypes.

## Author Contributions

**Mario Amatruda**: conceptualization, methodology, visualization, validation, formal analysis, writing–review and editing; investigation; project administration. **Juan Turati**: methodology, validation, visualization; formal analysis, investigation; project administration. **Josh Weiss**: methodology, validation, formal analysis, investigation, project administration. **Jorge Villavicencio**: methodology, validation, visualization, formal analysis, investigation, project administration. **Zhihong Chen**: methodology, validation, formal analysis. **Graham Britton**: methodology, validation, formal analysis. **Sam Horng**: methodology, validation, conceptualization, visualization; formal analysis, project administration, supervision, writing–original draft, writing–review and editing, investigation.

## Ethics Statement

Studies using mice were approved by the IACUC at the ISMMS and adhered to the American Veterinary Medical Association guidelines. The ISMMS has an Animal Welfare Assurance on file with the Office for Laboratory Animal Welfare (Assurance no. A3111‐01).

## Conflicts of Interest

The authors declare no conflicts of interest. Dr. Mario Amatruda was previously employed as a senior scientist at Biogen in 2023.

### Peer Review

The peer review history for this article is available at https://publons.com/publon/10.1002/brb3.70239.

## Supporting information




**Figure S1 Gating strategy for splenic leukocytes of the lymphoid lineage**. Multidimensional use of fluorescent surface antibodies targeted against CD45, CD3, CD4, CD8, and CD19 enabled the measurement and differentiation among helper and cytotoxic T cells and B cells.
**Figure S2 Gating strategy for splenic leukocytes of the myeloid lineage**. Multidimensional use of fluorescent surface antibodies targeted against CD45, CD11b, Siglec‐F, Ly6C, Ly6G, F4/80, and MHC‐II enabled the measurement and differentiation of neutrophils, dendritic cells, monocytes, macrophages, and eosinophils.
**Figure S3 Gating strategy for spinal cord leukocytes of the lymphoid lineage**. Multidimensional use of fluorescent surface antibodies targeted as above for splenic cells enabled the measurement and differentiation among helper and cytotoxic T and B cells as above.
**Figure S4 Gating strategy for spinal cord leukocytes of the myeloid lineage**. Multidimensional use of fluorescent surface antibodies targeted as above for splenic cells enabled the measurement and differentiation of neutrophils, dendritic cells, monocytes, macrophages, and eosinophils.
**Figure S5 In the absence of tamoxifen treatment, very low levels of spontaneous loxP recombination occur in splenic leukocytes of *Aldh1l1‐Cre/ERT2;ROSA* mice, whereas tamoxifen‐treatment is not sufficient to drive loxP recombination in splenic leukocytes of *Gfap‐Cre;ROSA* mice**. Control experiments demonstrated by flow cytometry very low levels of spontaneous tdTomato reporter expression in splenic CD45^+^ cells of non‐tamoxifen treated healthy *Aldh1l1‐Cre/ERT2;ROSA* mice (A), whereas tamoxifen treatment (tam) did not lead to tdTomato reporter expression in splenic CD45^+^ cells of *Gfap‐Cre;ROSA* mice (B) (mean 1.57% vs 0.06%, *p* = 0.53). Comparisons were made to healthy, tamoxifen‐treated *Aldh1l1‐Cre/ERT2;ROSA* mice (C) (mean 9.21%) and these comparisons reached statistical significance (D) (untreated *Aldh1l1‐Cre/ERT2;ROSA* vs. tam‐treated *Aldh1l1‐Cre/ERT2;ROSA*, *p* < 0.01, tam‐treated *Gfap‐Cre;ROSA* vs. tam‐treated *Aldh1l1‐Cre/ERT2;ROSA*, *p* < 0.005, *n* = 3 in both untreated *Aldh1l1‐Cre/ERT2;ROSA* and tam‐treated *Gfap‐Cre;ROSA* groups, *n* = 2 in tam‐treated *Aldh1l1‐Cre/ERT2;ROSA)*. Flow was performed 1.5–2 weeks after tamoxifen treatment. One‐way ANOVA with Tukey's multiple comparison test.
**Figure S6 Representative flow cytometry plot demonstrating increased fluorescence intensity of tdTomato^+^ CD45^+^ cells within the CNS compared to tdTomato^−^ CD45^+^ cells (astrocytes)**. In spinal cord flow cytometry experiments, tdTomato^+^ CD45^+^ cells demonstrate a higher fluorescence intensity than tdTomato**^−^** CD45^+^ cells (astrocytes) and thus are easily differentiated on both the CD45^+^ and tdTomato axes.
**Table S1** List of antibodies used for multidimensional flow cytometry experiments.
**Table S2** Primer sequences and product length for genotyping of *mGfap‐Cre*, *Aldh1l1‐Cre/ERT2*, *Rosa‐tdTomato* wild‐type and mutant alleles.

## Data Availability

The data that support the findings of this study are available from the corresponding author, upon reasonable request.
