# Supplementary material for: Aldh1l1‐Cre/ERT2 Drives Flox‐Mediated Recombination in Peripheral and CNS Infiltrating Immune Cells in Addition to Astrocytes During CNS Autoimmune Disease
Source: Brain Behav. 2025 Feb 5;15(2):e70239. doi: 10.1002/brb3.70239 (PMC11799068; doi:10.1002/brb3.70239)
Supplement: Supplementary file 1 — Figure S1 Gating strategy for splenic leukocytes of the lymphoid lineage. Multidimensional use of fluorescent surface antibodies targeted against CD45, CD3, CD4, CD8, and CD19 enabled the measurement and differentiation among helper and cytotoxic T cells and B cells. Figure S2 Gating strategy for splenic leukocytes of the myeloid lineage. Multidimensional use of fluorescent surface antibodies targeted against CD45, CD11b, Siglec‐F, Ly6C, Ly6G, F4/80, and MHC‐II enabled the measurement and differentiation of neutrophils, dendritic cells, monocytes, macrophages, and eosinophils. Figure S3 Gating strategy for spinal cord leukocytes of the lymphoid lineage. Multidimensional use of fluorescent surface antibodies targeted as above for splenic cells enabled the measurement and differentiation among helper and cytotoxic T and B cells as above. Figure S4 Gating strategy for spinal cord leukocytes of the myeloid lineage. Multidimensional use of fluorescent surface antibodies targeted as above for splenic cells enabled the measurement and differentiation of neutrophils, dendritic cells, monocytes, macrophages, and eosinophils. Figure S5 In the absence of tamoxifen treatment, very low levels of spontaneous loxP recombination occur in splenic leukocytes of Aldh1l1‐Cre/ERT2;ROSA mice, whereas tamoxifen‐treatment is not sufficient to drive loxP recombination in splenic leukocytes of Gfap‐Cre;ROSA mice. Control experiments demonstrated by flow cytometry very low levels of spontaneous tdTomato reporter expression in splenic CD45+ cells of non‐tamoxifen treated healthy Aldh1l1‐Cre/ERT2;ROSA mice (A), whereas tamoxifen treatment (tam) did not lead to tdTomato reporter expression in splenic CD45+ cells of Gfap‐Cre;ROSA mice (B) (mean 1.57% vs 0.06%, p = 0.53). Comparisons were made to healthy, tamoxifen‐treated Aldh1l1‐Cre/ERT2;ROSA mice (C) (mean 9.21%) and these comparisons reached statistical significance (D) (untreated Aldh1l1‐Cre/ERT2;ROSA vs. tam‐treated Aldh1l1‐Cre/ERT2;ROSA, p [file BRB3-15-e70239-s001.docx]

**Supplemental Figure 1: Gating strategy for splenic leukocytes of the lymphoid lineage.** Multidimensional use of fluorescent surface antibodies targeted against CD45, CD3, CD4, CD8, and CD19 enabled the measurement and differentiation among helper and cytotoxic T cells and B cells.

**Supplemental Figure 2: Gating strategy for splenic leukocytes of the myeloid lineage.** Multidimensional use of fluorescent surface antibodies targeted against CD45, CD11b, Siglec-F, Ly6C, Ly6G, F4/80 and MHC-II enabled the measurement and differentiation of neutrophils, dendritic cells, monocytes, macrophages, and eosinophils.

**Supplemental Figure 3: Gating strategy for spinal cord leukocytes of the lymphoid lineage.** Multidimensional use of fluorescent surface antibodies targeted as above for splenic cells enabled the measurement and differentiation among helper and cytotoxic T cells and B cells as above.

**Supplemental Figure 4: Gating strategy for spinal cord leukocytes of the myeloid lineage.** Multidimensional use of fluorescent surface antibodies targeted as above for splenic cells enabled the measurement and differentiation of neutrophils, dendritic cells, monocytes, macrophages, and eosinophils.

**Supplemental Figure 5: In the absence of tamoxifen treatment, very low levels of spontaneous loxP recombination occur in splenic leukocytes of *Aldh1l1-Cre/ERT2;ROSA* mice while tamoxifen-treatment is not sufficient to drive loxP recombination in splenic leukocytes of *Gfap-Cre;ROSA* mice.** Control experiments demonstrated by flow cytometry very low levels of spontaneous tdTomato reporter expression in splenic CD45^+^ cells of non-tamoxifen treated healthy *Aldh1l1-Cre/ERT2;ROSA* mice (A) while tamoxifen treatment (tam) did not lead to tdTomato reporter expression in splenic CD45^+^ cells of *Gfap-Cre;ROSA* mice (B) (mean 1.57% vs 0.06%, p=0.53). Comparisons were made to healthy, tamoxifen-treated *Aldh1l1-Cre/ERT2;ROSA* mice (C) (mean 9.21%) and these comparisons reached statistical significance (D) (untreated *Aldh1l1-Cre/ERT2;ROSA* vs. tam-treated *Aldh1l1-Cre/ERT2;ROSA*, p<0.01, tam-treated *Gfap-Cre;ROSA* vs. tam-treated *Aldh1l1-Cre/ERT2;ROSA*, p<0.005, n=3 in both untreated *Aldh1l1-Cre/ERT2;ROSA* and tam-treated *Gfap-Cre;ROSA* groups, n=2 in tam-treated *Aldh1l1-Cre/ERT2;ROSA)*. Flow was performed 1.5-2 weeks after tamoxifen treatment. One-way ANOVA with Tukey’s multiple comparison test.

**Supplemental Figure 6: Representative flow cytometry plot demonstrating increased fluorescence intensity of tdTomato^+^ CD45^+^ cells within the CNS compared to tdTomato^-^ CD45^+^ cells (astrocytes).** In spinal cord flow cytometry experiments, tdTomato^+^ CD45^+^ cells demonstrate a higher fluorescence intensity than tdTomato^-^ CD45^+^ cells (astrocytes) and thus are easily differentiated on both the CD45^+^ and tdTomato axes.

**Supplemental Table 1**

List of antibodies used for multidimensional flow cytometry experiments.

**Supplemental Table 2**

Primer sequences and product length for genotyping of *mGfap-Cre*, *Aldh1l1-Cre/ERT2*, *Rosa-tdTomato* wild-type and mutant alleles.

**Supplemental Figure 1**


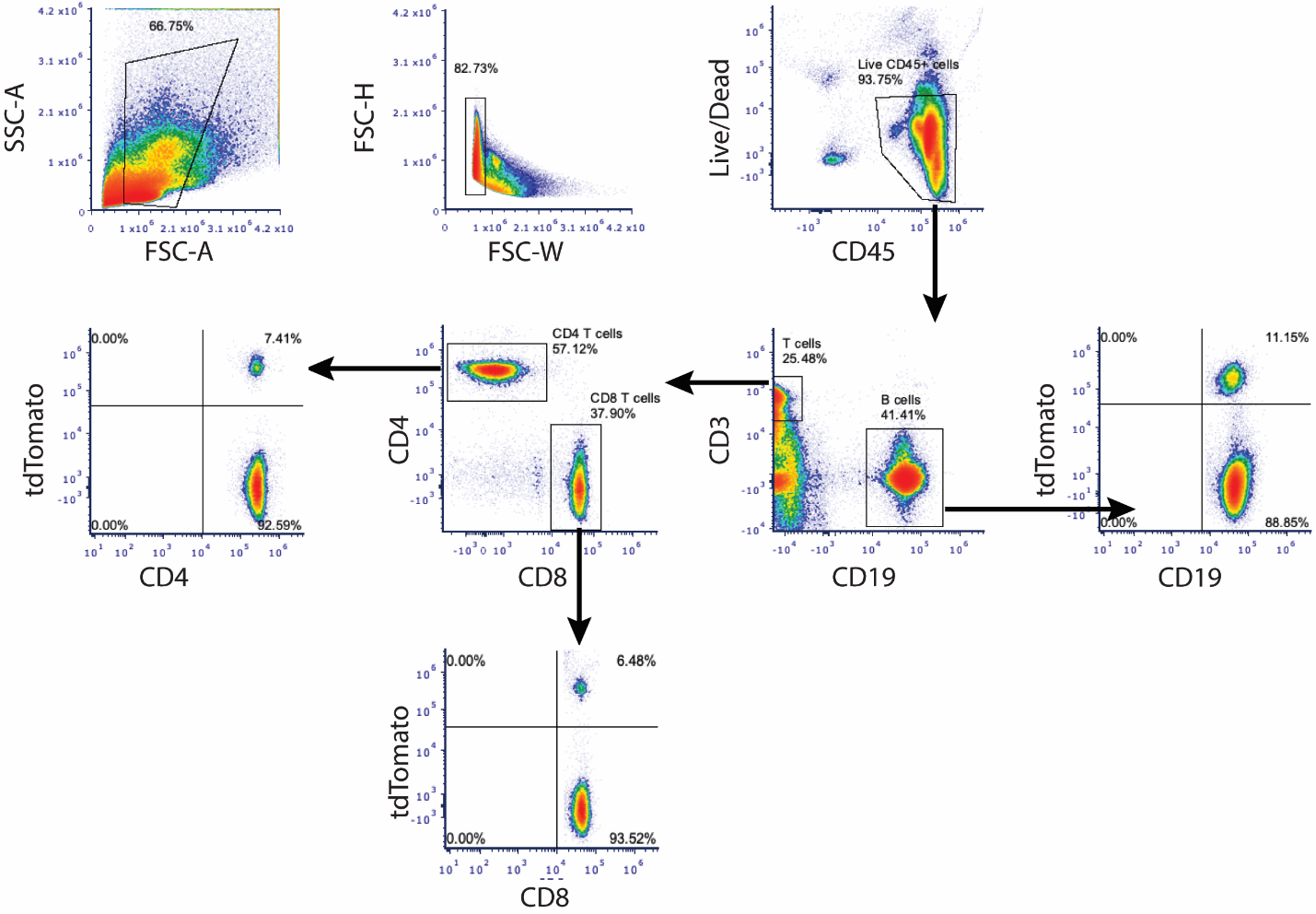


**Supplemental Figure 2**


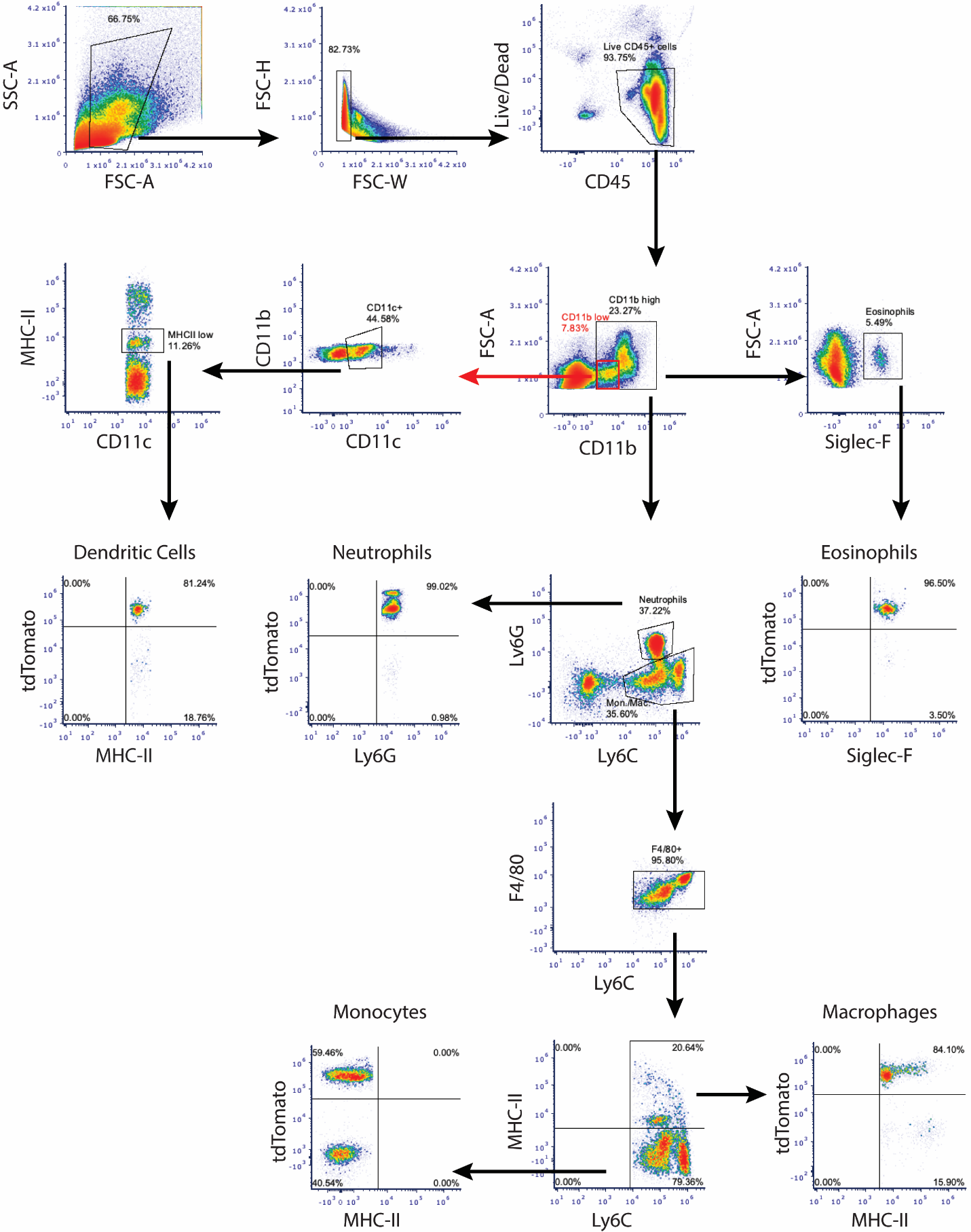


**Supplemental Figure 3**

**
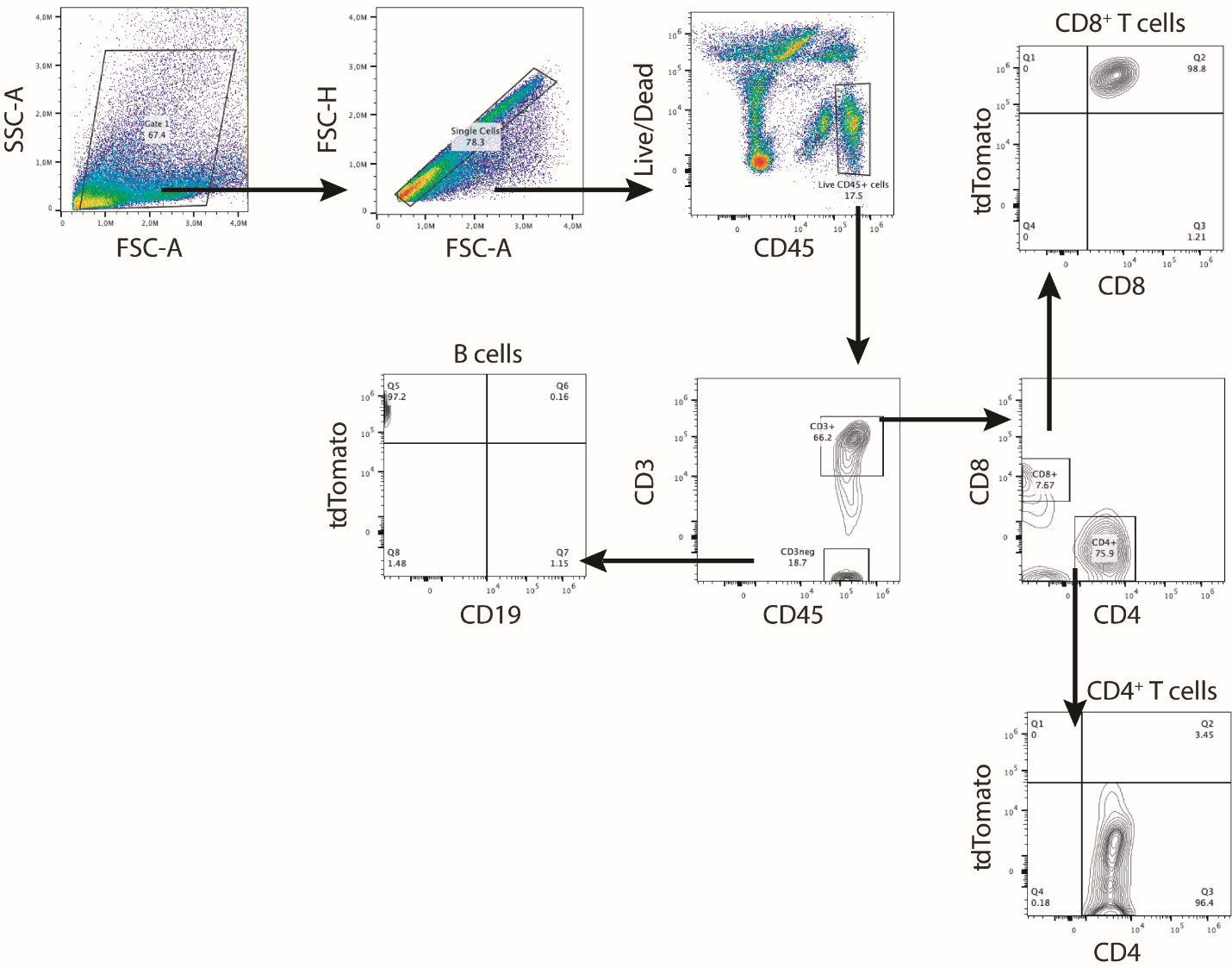
**

**Supplemental Figure 4**

**
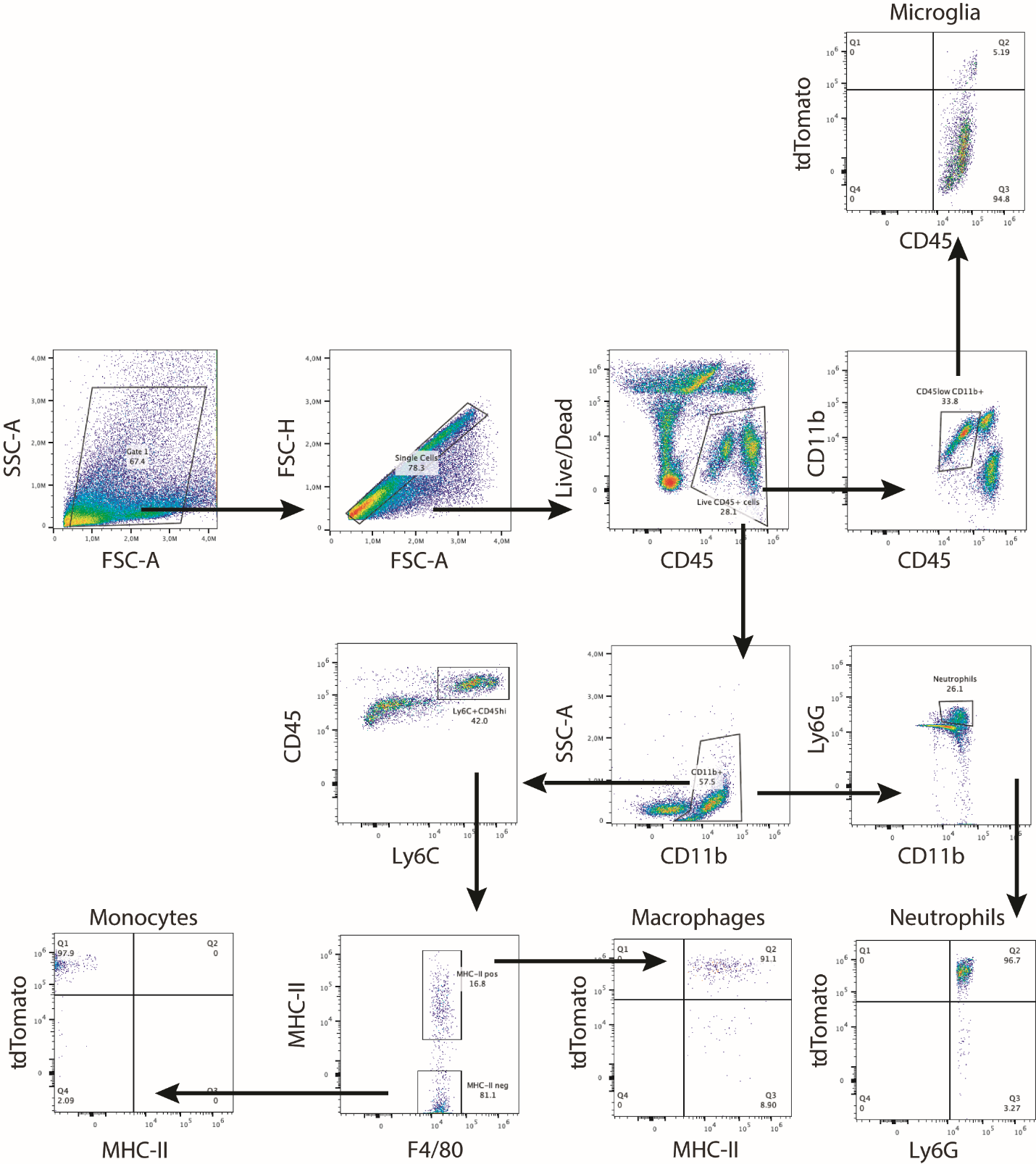
**

**Supplemental Figure 5**

**
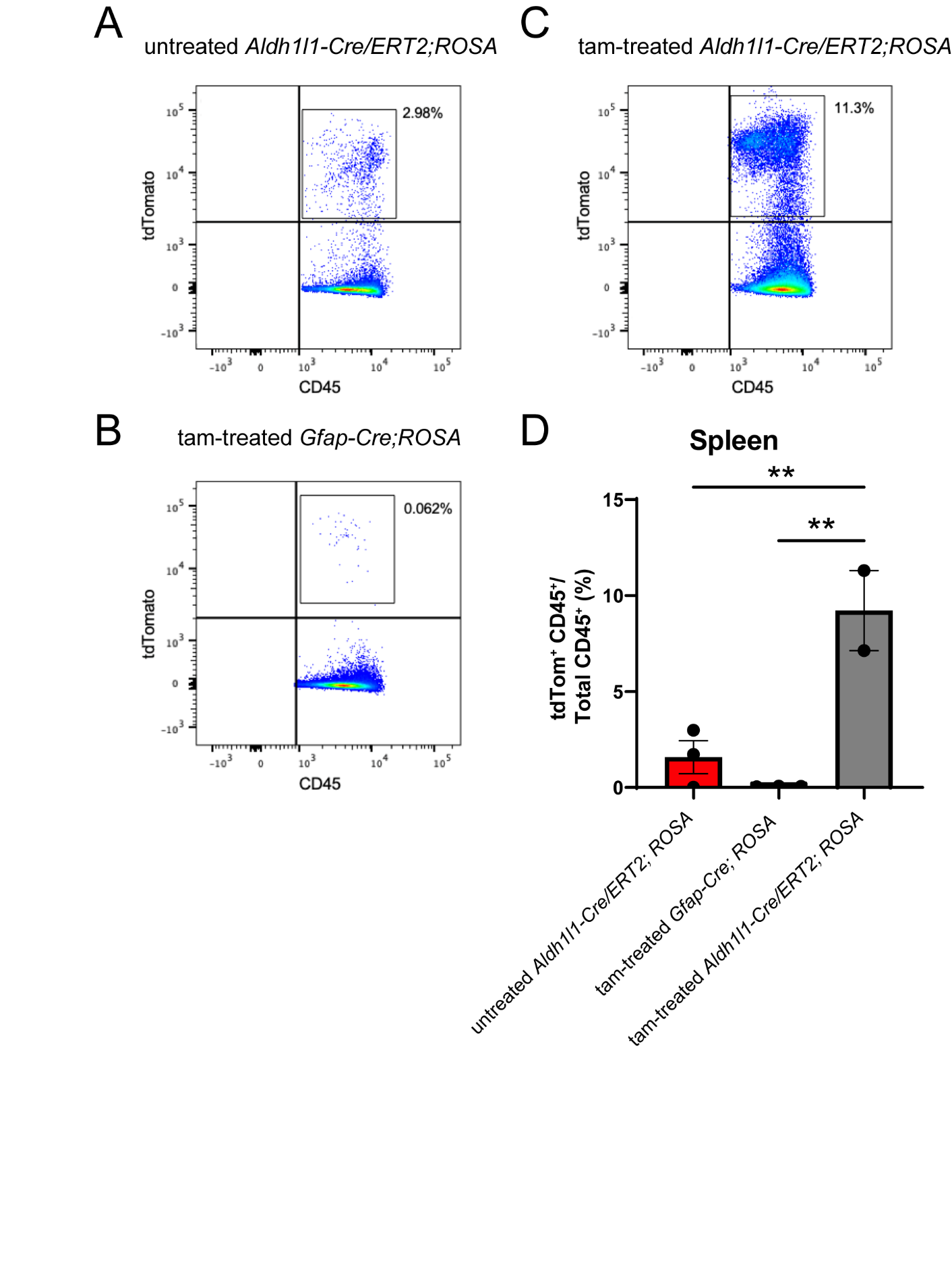
**

**Supplemental Figure 6**

**
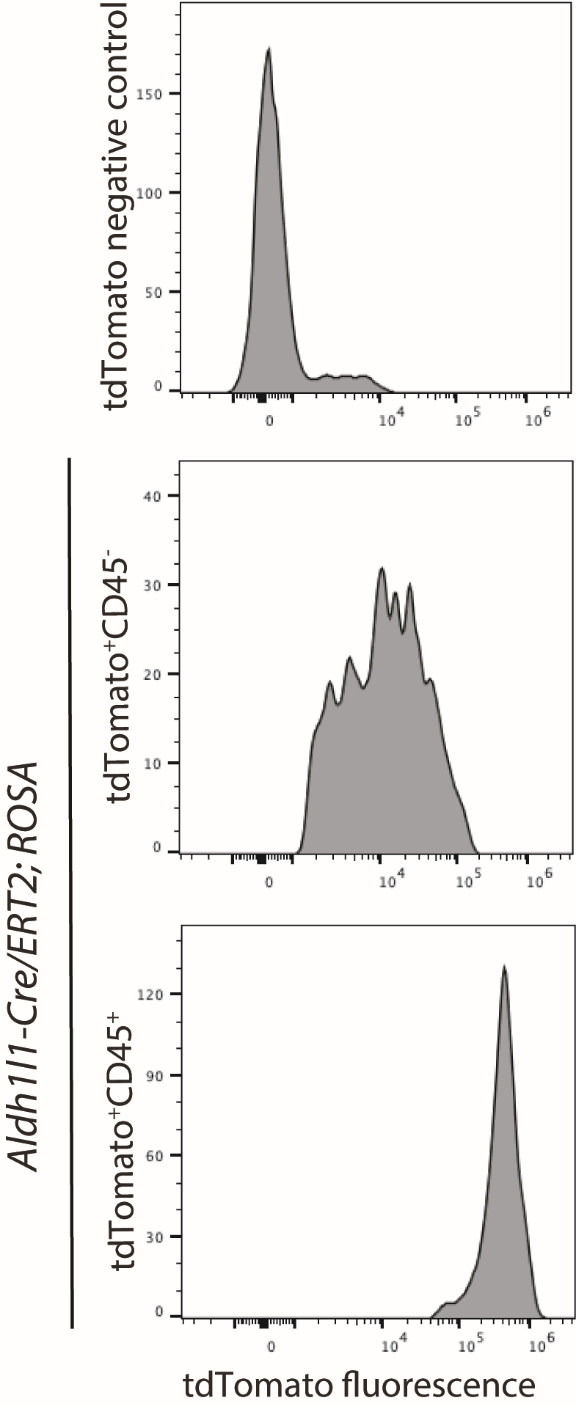
**

**Supplemental Table 1**

| **Marker** | **Fluorophore** | **Clone** | **Supplier** | **Cat#** | **Working dilution** |
| --- | --- | --- | --- | --- | --- |
| tdTomato | tdTomato | N/A | N/A | N/A | N/A |
| Zombie NIR | similar to APC/Cy7 | N/A | Biolegend | 423105 | 1:1000 |
| CD45 | BV750 | 30-F11 | Biolegend | 103157 | 1:200 |
| CD11b | PerCp-Cy5.5 | M1/70 | Biolegend | 101227 | 1:1000 |
| Siglec-F | AF647 | E50-2440 | BD | 562680 | 1:100 |
| CD3 | PE | 17A2 | Biolegend | 100205 | 1:200 |
| Ly6G | BV570 | 1A8 | Biolegend | 127629 | 1:100 |
| Ly6C | FITC | FITC | Biolegend | 128006 | 1:100 |
| CD19 | BV711 | 6D5 | Biolegend | 115555 | 1:100 |
| MHC-II | Pacific Blue | M5/114.15.2 | Biolegend | 107620 | 1:500 |
| CD4 | PE-Cy7 | RM4-5 | Biolegend | 100527 | 1:300 |
| F4/80 | BV605 | BM8 | Biolegend | 123133 | 1:100 |
| CD8 | Pacific Orange | 5H10 | Thermo | MCD0830 | 1:50 |

**Supplemental Table 2**

| **Genotype** | **Forward Primer** | **Reverse Primer** | **Band Length** |
| --- | --- | --- | --- |
| ***mGfap-Cre* (73-12)** | 5'-ACCAGCCAGCTATCAACTC-3' | 5'-TATACGCGTGCTAGCGAAGATCTCCATCTTCCAGCAG-3' | 350 bp |
| ***Aldh1l1-Cre/ERT2*** | 5'-CTTCAACAGGTGCCTTCCA-3' | 5'-GGCAAACGGACAGAAGCA-3' | 200 bp |
| ***Rosa-tdTomato***  ***Wild type*** | 5'-CTGTTCCTGTACGGCATGG-3' | 5'-GGCATTAAAGCAGCGTATCC-3' | 320 bp |
| ***Rosa-tdTomato***  ***Mutant*** | 5'-AAGGGAGCTGCAGTGGAGTA-3' | 5'-CCGAAAATCTGTGGGAAGTC-3' | 200 bp |
